# Amenable mortality as a performance indicator of Italian health-care services

**DOI:** 10.1186/1472-6963-12-310

**Published:** 2012-09-10

**Authors:** Maria P Fantini, Jacopo Lenzi, Giuseppe Franchino, Cristina Raineri, Alessandra Burgio, Luisa Frova, Gianfranco Domenighetti, Walter Ricciardi, Gianfranco Damiani

**Affiliations:** 1 Department of Medicine and Public Health, Alma Mater Studiorum, University of Bologna, Bologna, 40126, Italy; 2 Health and Assistance Unit, Italian National Institute of Statistics, ISTAT, Rome, Italy; 3 Institute of Public Communication and Institute of Microeconomics and Public Economics, University of Lugano, Lugano, 6900, Switzerland; 4 Department of Public Health, Università Cattolica del Sacro Cuore, Rome, Italy

**Keywords:** Amenable mortality, Health-care services performance, Socioeconomic status, Gender

## Abstract

**Background:**

Mortality amenable to health-care services (‘amenable mortality’) has been defined as “premature deaths that should not occur in the presence of timely and effective health care” and as “conditions for which effective clinical interventions exist.” We analyzed the regional variability in health-care services using amenable mortality as a performance indicator. Convergent validity was examined against other indicators, such as health expenditure, GDP per capita, life expectancy at birth, disability-free life expectancy at age 15, number of diagnostic and laboratory tests per 1,000 inhabitants, and the prevalence of cancer and cardiovascular diseases.

**Methods:**

Amenable mortality rate was calculated as the average annual number of deaths in the population aged 0–74 years per 100,000 inhabitants, and it was then stratified by gender and region. Data were drawn from national mortality statistics for the period 2006–08.

**Results:**

During the study period (2006–08), the age-standardized death rate (SDR) amenable to health-care services in Italy was 62.6 per 100,000 inhabitants: 66.0 per 100,000 for males and 59.1 per 100,000 for females. Significant regional variations ranged from 54.1 per 100,000 inhabitants in Alto Adige to 76.3 per 100,000 in Campania. Regional variability in SDR was examined separately for male and females. The variability proved to be statistically significant for both males and females (males: Q-test = 638.5, p < 0.001; females: Q-test = 700.1, p < 0.001). However, among men, we found a clear-cut divide in SDR values between Central and Southern Italy; among women, this divide was less pronounced. Amenable mortality was negatively correlated with life expectancy at birth for both genders (male: r = −0.64, *p* = 0.002; female: r = −0.88, *p* <0.001) and with disability-free life expectancy at age 15 (male: r = −0.70, *p* <0.001; female: r = −0.67, *p* <0.001). Amenable mortality displayed a statistically significant negative relationship with GDP per capita, the quantity of diagnostic and laboratory tests per 1,000 inhabitants, and the prevalence of cancer.

**Conclusions:**

Amenable mortality shows a wide variation across Italian regions and an inverse relationship with life expectancy and GDP per capita, as expected.

## Background

The performance of health-care systems in terms of maximizing population health has been a major concern of policy makers [1-5]. Explicit frameworks defining the goals of a health system, against specific outcomes and performance indicators are required [6].

In recent years, the concept of amenable mortality has been used as a proxy for performance of health-care systems by Nolte and McKee [7-9] and Tobias and Yeh [10]. These authors took the term “amenable mortality”, developed by European researchers in the 1980s and 1990s [8,11], to assess the quality and performance of health systems over time [8,9].

Mortality amenable to health-care services (hereafter amenable mortality) has been defined as “premature deaths that should not occur in the presence of timely and effective health care” [9] and as “conditions for which effective clinical interventions exist” [10]. As might be expected, the correlation between amenable mortality rates and life expectancy is high since amenable mortality is, by definition, included in overall mortality, even if amenable mortality rates may differ in countries with similar life expectancy at birth [11]. Furthermore, amenable mortality also correlates with disability-adjusted life expectancy with better face validity [7].

Nolte and McKee carried out a comprehensive study [9] of amenable mortality in 19 Organization for Economic Co-operation and Development (OECD) countries between 1997–98 and 2002–03, and they found a clear decline in amenable mortality in all the countries investigated. In that study, Italy showed a reduction in amenable mortality from 88.7 per 100,000 (1997–98) to 74.0 per 100,000 (2002), and in 2002 it ranked fifth among the 19 OECD countries.

Another recent study [11] has provided estimates of amenable mortality for a large set (31) of OECD countries, and it assessed the sensitivity of this indicator by comparing the two widely used lists (those of Nolte and McKee [9] and Tobias and Yeh [10]). Results published by the OECD [11] show that in 2007 age-standardized amenable mortality rates ranged from 60 to 200 deaths per 100,000 in the OECD countries. The above two lists provided similar results for most countries. Eastern European countries and Mexico had the highest rates; Japan, France, Italy, Sweden, and Iceland had the lowest ones. Mortality rates for Italy (2006 data) ranged from 65 deaths per 100,000 (Nolte and McKee’s list) to 71 per 100,000 (Tobias and Yeh’s list). Italy ranked third in both lists.

Amenable mortality is also a useful indicator for measuring the performance of health-care systems. As a strategy to deal with shrinking resources while increasing local government accountability, efficiency, quality, and innovation in the health-care sector, the process of decentralization of powers from national to regional levels has been widely implemented across European health-care systems [12]. Therefore, the distribution of powers between central and regional levels as well as their respective roles in funding and providing health-care services is crucial.

In Italy, the central government is responsible for national health planning and annual funding. It also has the exclusive power to set the so-called essential levels of care (Livelli Essenziali di Assistenza; LEAs), an explicit, publicly funded health-benefit package to which all citizens are entitled. Regions have virtually exclusive responsibility for the organization and administration of publicly financed health care [13]. Therefore, monitoring the performance of regional levels of the health-care system has become of increasing interest in policy decision making.

The aim of this study was twofold. The first was to analyze the regional variability in health-care services using amenable mortality as a performance indicator. The second was to examine the convergent validity of amenable mortality against other indicators, such as health expenditure, GDP per capita, life expectancy at birth, disability-free life expectancy at age 15, number of diagnostic and laboratory tests per 1,000 inhabitants, and the prevalence of cancer and cardiovascular diseases.

## Methods

A cross-sectional study was carried out on individual data from national mortality statistics for the period 2006–08 using data from the Italian National Institute of Statistics (ISTAT), where the causes of death are coded using the ICD-10 classification. Nolte and McKee [7-9] and Tobias and Yeh [10] prepared two different lists of causes of death amenable to health-care [7]. These two lists were used by OECD to generate estimates of amenable mortality for 31 countries [11]. After reviewing the two sets of estimates of amenable mortality for OECD countries provided by Nolte and McKee’s and Tobias and Yeh’s lists, we decided to choose Nolte and McKee’s list because it offers on average more conservative figures.

Amenable mortality rate was calculated as the average annual number of deaths over the population aged 0–74 years per 100,000 inhabitants, and it was then stratified by gender, region (19 regions and the two autonomous provinces of Trentino and Alto Adige) and 10 disease categories defined by Gay et al. in an OECD report (see Additional file 1: Table S1) [11]. We computed age-standardized death rates (SDRs) using the 2005 OECD population as the standard population.

Forest plots and Cochrane’s Q-test were used to compare the regional SDRs with the Italian average. We computed 95% confidence intervals using Chiang’s normal approximation to Poisson distribution [14]. Regional SDRs for specific disease categories were plotted in relation to the Italian average using radar graphs.

We examined the relationship between amenable mortality and life expectancy at birth as well as disability-free life expectancy at age 15, using linear regression models stratified by gender as suggested in the literature [7,11]. Disability-free life expectancy at age 15 indicates the expected number of healthy life-years. Linear regression models were also used to analyze the relationship between regional SDRs and public health expenditure per capita, GDP per capita, number of diagnostic and laboratory tests per 1,000 inhabitants, cancer prevalence, and prevalence of cardiovascular diseases.

Disability-free life expectancy, cancer prevalence, and prevalence of cardiovascular diseases were drawn from the ISTAT Multiscope National Survey [15]. Public health expenditure and GDP per capita were drawn from Rapporto Osservasalute for 2011 [16]. The number of diagnostic and laboratory tests was taken from the Ministry of Health data on health-care activities [17].

Results are provided by regions. Northern Italy includes Piedmont, Aosta Valley, Lombardy, Alto Adige, Trentino, Veneto, Friuli-Venezia Giulia, Liguria and Emilia-Romagna; Central Italy includes Tuscany, Umbria, Marche and Lazio; Southern Italy includes Abruzzo, Molise, Campania, Puglia, Basilicata, Calabria, Sicily, and Sardinia. Statistical analyses were performed using Stata 11 [18].

## Results

### Differences in amenable mortality among Italian regions

**Figure 1:**
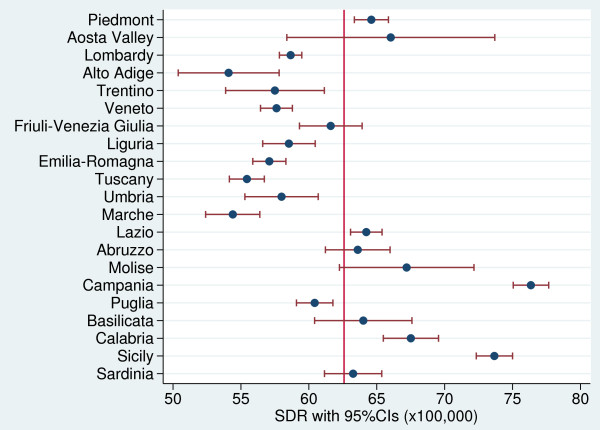
Forest plot showing the regional SDRs (with 95% confidence intervals [CIs]) in relation to the Italian average (red line) for the years 2006–08.

**Figure 2:**
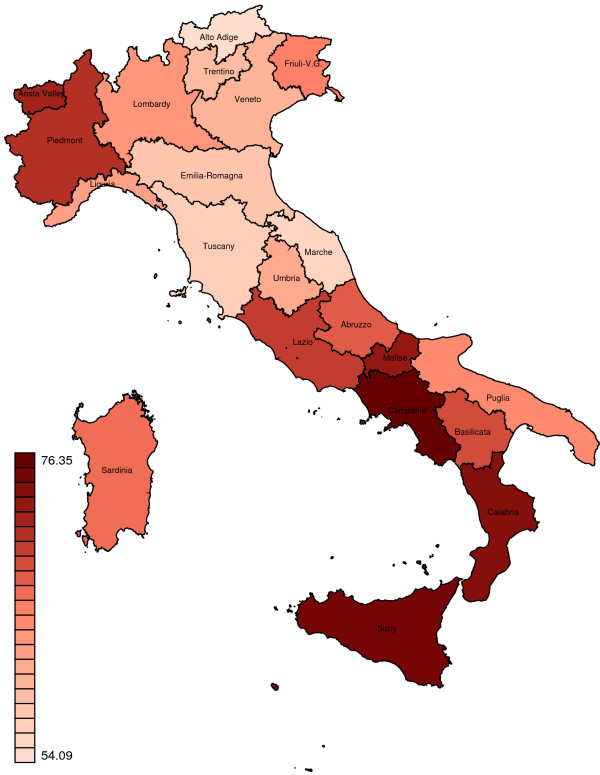
Geographical distribution of regional SDRs in Italy for the years 2006–08.

During the study period (2006–08), the SDR in Italy was 62.6 per 100,000 inhabitants: 66.0 per 100,000 for males and 59.1 per 100,000 for females. Figures 1 and 2 shows the regional distribution of SDRs. A statistically significant regional variation was found, with a range from 54.1 per 100,000 in Alto Adige to 76.3 per 100,000 in Campania. Specifically, results indicate that for five regions (Piedmont, Lazio, Campania, Calabria, and Sicily), SDRs were statistically significantly higher than the national average, whereas for six regions (Aosta Valley, Friuli-Venezia Giulia, Abruzzo, Molise, Basilicata, and Sardinia) the rates did not differ statistically significantly from the national average. Finally, in ten regions (Lombardy, Trentino, Alto Adige, Veneto, Liguria, Emilia-Romagna, Tuscany, Umbria, Marche, and Puglia), SDRs were statistically significantly lower than the national average. Southern Italy generally had higher SDRs than Northern Italy, except for Piedmont and Puglia.

**Figure 3:**
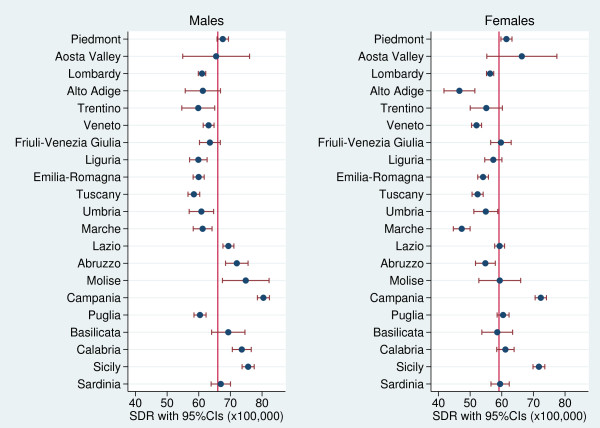
Forest plots showing the regional SDRs (with 95% CIs) by gender in relation to the Italian average (red line) for the years 2006–08.

Figure 3 shows the regional distribution of SDRs by gender. The regional variability proved to be statistically significant for both males and females (males: Q-test = 638.5, p < 0.001; females: Q-test = 700.1, p < 0.001). However, among men, we found a clear-cut divide in SDR values between Central and Southern Italy; specifically, in general 95% CIs of SDRs exceeded the national value in Southern regions, while in Northern and Central regions were lower than the national average. Among women, the regional variability proved to be greater than among men, but 95% CIs of regional SDRs did not reveal a clear-cut geographical pattern. Campania had the highest gender-specific SDRs.

**Figure 4:**
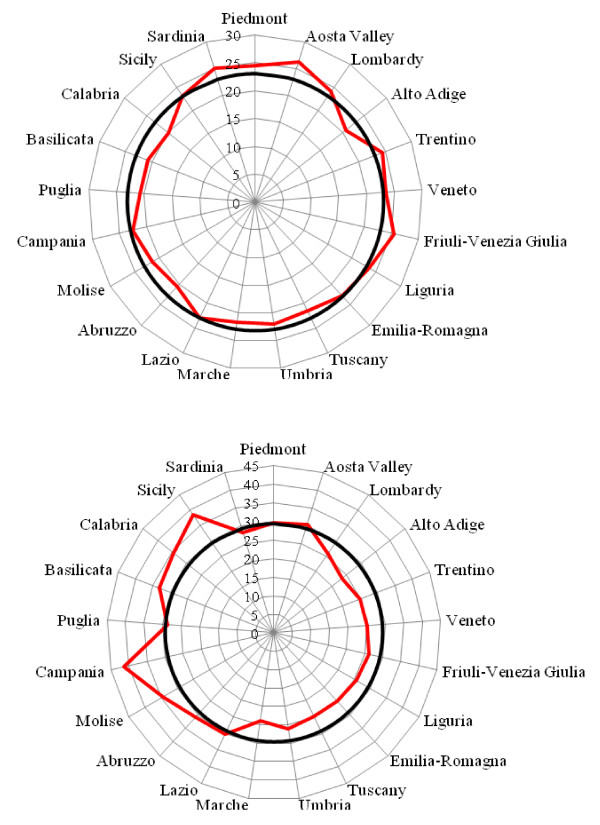
**a. Radar plot showing the regional SDRs for cancers in relation to the Italian average (black line) for the years 2006–08.b**. Radar plot showing the regional SDRs for cardiovascular diseases in relation to the Italian average (black line) for the years 2006–08.

Cancer and cardiovascular diseases were the leading causes of amenable mortality (23.0 and 29.3 per 100,000) and accounted, respectively, for 36.5% and 49.0% of overall amenable mortality. Figure 4a, b shows the radar plots of the regional SDRs compared with the national average for these diseases. Though regional SDRs for cancer exhibited a limited departure from the national average, regional SDRs for cardiovascular diseases were above the mean in Southern Italy and below the mean in Central and Northern Italy.

### Convergent validity of the indicator

**Figure 5:**
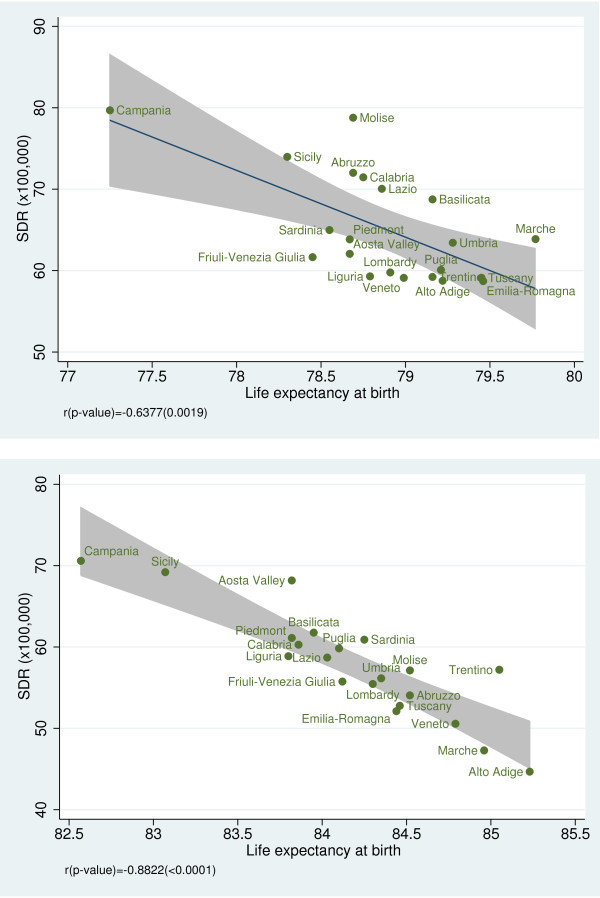
**a. Amenable mortality vs. life expectancy at birth (males) for the year 2008 (males).** Data source: Health for All 2011 [19]. **b**. Amenable mortality vs. life expectancy at birth (females) for the year 2008. Data source: Health for All 2011 [19].

**Figure 6:**
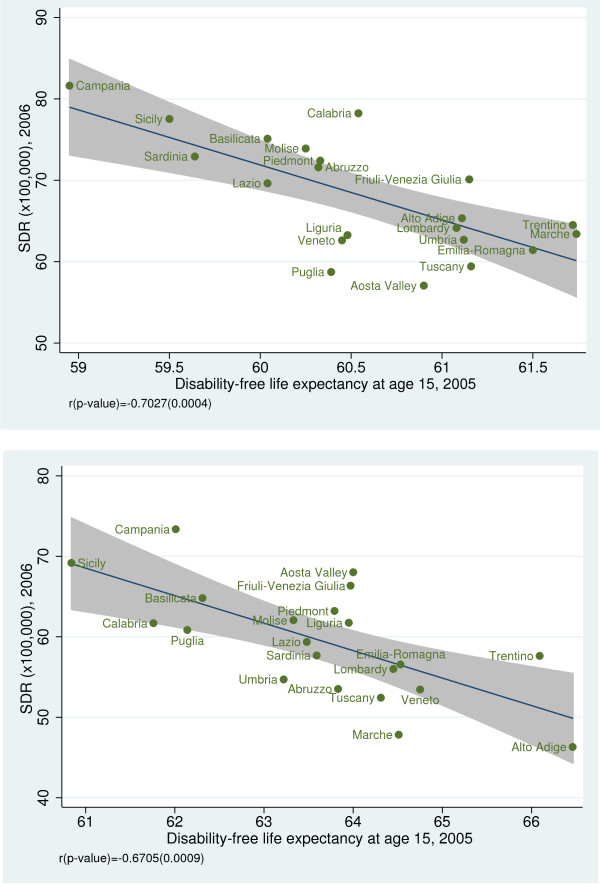
**a.Amenable mortality vs. disability-free life expectancy at age 15 (males).** Data source: Indagine Istat su condizioni di salute e ricorso ai servizi sanitari 2004–2005 [15]. **b**. Amenable mortality vs. disability-free life expectancy at age 15 (females). Data source: Indagine Istat su condizioni di salute e ricorso ai servizi sanitari 2004–2005 [15].

**Figure 7:**
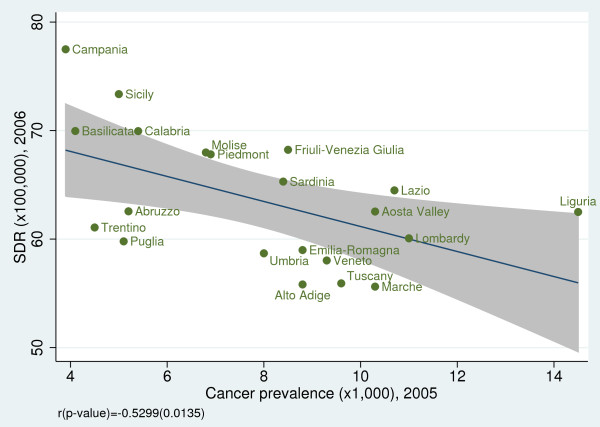
**Amenable mortality vs. cancer prevalence; cancer includes all types of malignant tumors.** Data source: Indagine Istat su condizioni di salute e ricorso ai servizi sanitari 2004–2005 [15].

**Figure 8:**
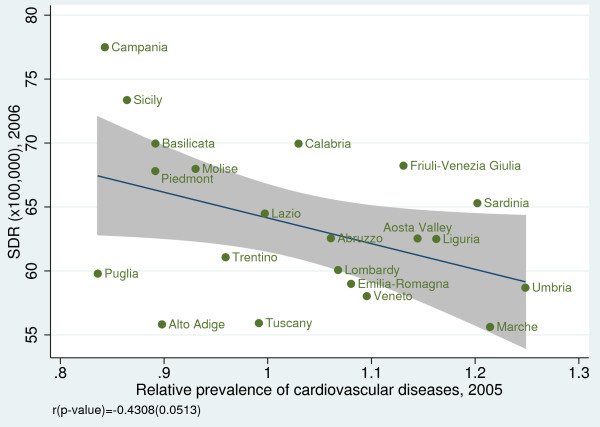
**Amenable mortality vs. relative prevalence of cardiovascular diseases, including stroke, hypertension, acute myocardial infarction, and angina pectoris.** Data source: Indagine Istat su condizioni di salute e ricorso ai servizi sanitari 2004–2005 [15].

To investigate the convergent validity of amenable mortality, we examined the association between SDRs and some health indicators. We observed a statistically significant negative correlation between SDR and life expectancy at birth for both genders (male: r = −0.64, *p* = 0.002; female: r = −0.88, *p* < 0.001) (Figure 5a, b) [19] and between SDR and disability-free life expectancy at age 15 (male: r = −0.70, *p* < 0.001; female: r =−0.67, *p* < 0.001) (Figure 6a, b). Furthermore, we found a statistically significant inverse relationship between SDR and the regional prevalence of cancer (r = −0.53; *p* = 0.013) (Figure 7) and a non statistically significant negative correlation between SDR and the prevalence of cardiovascular diseases (r = −0.43; *p* = 0.051) (Figure 8).

### Relationship of amenable mortality with socioeconomic indicators

**Figure 9:**
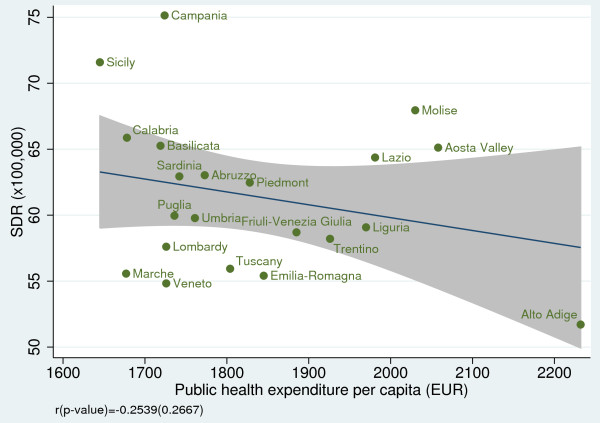
**Amenable mortality vs. public health expenditure per capita for the year 2008.** Data source: Rapporto Osservasalute 2011 [16].

**Figure 10:**
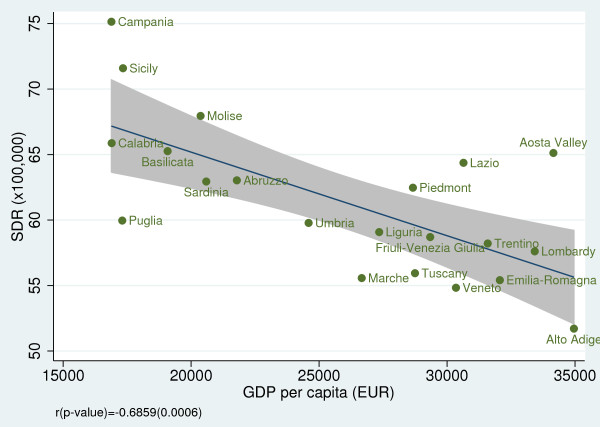
**Amenable mortality vs. GDP per capita for the year 2008.** Data source: Rapporto Osservasalute 2011 [16].

**Figure 11:**
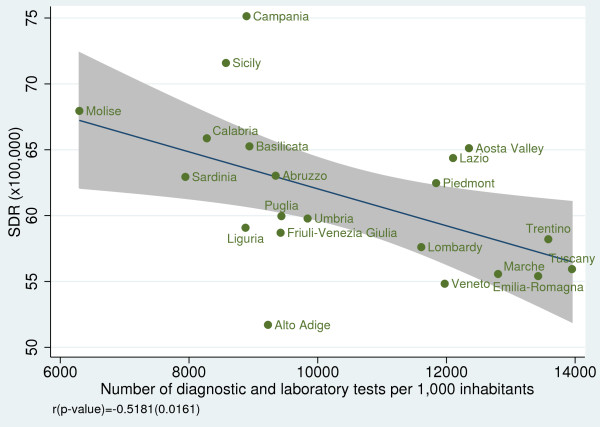
**Amenable mortality vs. number of diagnostic and laboratory tests per 1,000 inhabitants for the year 2008.** Data source: Ministero della Salute – Sistema Informativo Sanitario 2008 [17].

When we examined the effect of socioeconomic and resource-consumption indicators on SDRs, we found a non statistically significant association between SDR and public health spending per capita (r = −0.25; *p =* 0.267) (Figure 9); however, we found a strong negative correlation between SDR and GDP per capita (r = −0.69; *p <* 0.001) (Figure 10) and between SDR and number of diagnostic and laboratory tests per 1,000 inhabitants (r = −0.52; *p =* 0.016) (Figure 11).

## Discussion

Evidence from the most recent studies indicates that amenable mortality can be used to assess health-care performance across countries over time. Italy showed the third-lowest amenable mortality in 2005 among 31 OECD countries, indicating a good performance of health-care services [11].

Our study shows that for the period 2006–08, the SDR was 62.6 per 100,000 inhabitants, though there were regional differences with a clear-cut divide being evident between Central and Southern Italy. In Italy, disparities in the geographical distribution have been documented for educational level (percentage of the population with only elementary education: 22.5% in the North and 27.2% in the South) [20], income (family average net income: 37,440 euros in the North and 27,971 euros in the South) [21], and poverty (incidence of relative poverty: 4.9% in the North and 23.0% in the South) [22]. With regard to health-care supply, Southern regions have a smaller number of hospital beds, more private facilities, and a poorer endowment of advanced medical equipment: for example, Northern regions had 9 MRI scanners per million inhabitants in 2007 compared with 6.3 in Southern regions [23]. Our results are consistent with evidence from the literature, suggesting that the quality of care is better in Northern than in Southern Italian regions in terms of percentage of inappropriate hospital admissions [24], proportion of surgical interventions implemented within 48 hours of hospital admission for elderly patients with hip fractures [17,25], and adherence to breast cancer screening programs [26].

Moreover, the average SDR was lower in women (mean, 59.1; range, 46.6–72.3 per 100,000) than in men (mean, 66.0; range, 58.5–80.4 per 100,000). This may result from women’s higher life expectancy at birth and gender-specific help-seeking patterns. The divergence in SDR values between Central and Southern Italy was more evident among men than in women. Pinkhasov et al. [27] found different health-service utilization patterns among males and females, with the latter showing greater alacrity in accessing health-care services. These gender-specific attitudes may have an impact on morbidity, mortality, and life expectancy.

In our study, cancer and cardiovascular diseases proved to be the leading causes of amenable mortality, accounting for 85.5% of the overall indicators. A greater regional variability was found for cardiovascular diseases than for cancer. This finding may suggest that Nolte and McKee’s list is especially sensitive to conditions for which effective and appropriate health care is essential, such as cardiovascular diseases.

The convergent validity of amenable mortality with some health indicators was partially supported by a high negative correlation between SDR and life expectancy at birth and disability-free life expectancy at age 15 for both genders.

Our study shows that amenable mortality is related to GDP per capita, but not to health expenditure per capita. A possible interpretation of this finding is that SDR is influenced by organizational and care delivery models and different priority settings—not by the amount of dedicated resources. Recently, Nagy et al. [28] also reported a positive association between amenable mortality and deprivation status in both genders.

Several caveats should be noted in the use of amenable mortality as an outcome indicator of health-system performance. First, it is important to note that the categorization of a condition as amenable is essentially based on a judgment about the effectiveness of medical interventions in treating different conditions and preventing death [9]. Furthermore, the selection of causes of death that are “amenable to health care” is time-dependent: technological progress constantly increases the opportunities to prevent premature deaths through secondary prevention and treatments. Therefore, the list of causes of death that are amenable to health care changes over time and needs to be regularly updated [11]. However, our investigation was over a relatively short period of time (2006–08), during which there was probably no major change in this trend.

Moreover, we chose to use Nolte and McKee’s list of causes of death amenable to health-care. Unlike Tobias and Yeh’s list, Nolte and McKee’s list included among death causes adverse events to patients during surgical and medical care, which are strongly related to quality of care. Last, Tobias and Yeh’s list included among the death causes chronic obstructive pulmonary disease (age >45 years), whose prognosis is more related to lifestyle than to health care, and bladder and thyroid cancer, where treatment and surgical interventions are moderately effective.

Second, the prevalence of diseases whose deaths are amenable to health-care may vary across regions. For instance, if the prevalence of cancer is substantially higher in one region, this region will need to devote more resources to avoid deaths from this disease category [11]. To address this issue, we analyzed the relationship between amenable mortality and disease prevalence for cancer and cardiovascular diseases. We found evidence that amenable mortality displays a negative relationship with the prevalence of cancer. This may suggest that when this specific condition is common, health services become more experienced and effective in treating the condition.

Third, health-service performance may depend on the quantity of resources available to provide effective interventions. However, as previously noted, we discovered no relationship between SDR and per capita public health expenditure.

Keeping these limitations in mind, the present study provides, in the context of decentralization of powers in health care, an easily calculated, valid indicator for monitoring the performance of health-care systems as a basis for evidence-based policy decision making. This indicator could be useful not only for a comparison between systems but also to detect variations in a health-care system over time. And the indicator could be a positive element in continuous efforts to improve the policies, organization and quality in a decentralized health-care system.

## Conclusions

Our results highlight that, in line with other health-care performance indicators, amenable mortality is lower in North and Central regions of Italy than in Southern regions. We argue that this indicator can be used to inform policy decision-making processes in decentralized health-care systems and monitor their effectiveness and equity. Amenable mortality exhibits an inverse relationship with life expectancy, prevalence of cancer and cardiovascular diseases, and socioeconomic and resource consumption indicators. Further investigation is warranted to analyze the trend of SDRs at a subnational level over time.

## Competing interests

The authors declare that they have no competing interests.

## Authors’ contributions

MPF, GDa, and WR contributed to the conception of this paper. MPF and GDa conceived the study design; LF and AB provided data sources and participated in the study design. GF provided the acquisition of data; JL and CR conceived the statistical methodology and performed statistical analyses. GDa, MPF, GF, and JL drafted the manuscript. MPF and JL had full access to all of the data in the study and take responsibility for the integrity of the data and accuracy of the data analysis. GDo critically revised the draft and contributed to the final writing of the paper. All authors read and approved the final manuscript.

## Pre-publication history

The pre-publication history for this paper can be accessed here:

http://www.biomedcentral.com/1472-6963/12/310/prepub

## Supplementary Material

Additional file 1 **Table S1.** Nolte and McKee’s list of causes of death arranged into 10 disease categories. Click here for file
