# Supplementary material for: Amenable mortality as a performance indicator of Italian health-care services
Source: BMC Health Serv Res. 2012 Sep 10;12:310. doi: 10.1186/1472-6963-12-310 (PMC3506466; doi:10.1186/1472-6963-12-310)
Supplement: Additional file 1 — Table S1. Nolte and McKee’s list of causes of death arranged into 10 disease categories. [file 1472-6963-12-310-S1.doc]

Additional file 1: Table S1. Nolte and McKee’s list of causes of death arranged into 10 disease categories

| **10 disease categories in OECD report**[11] | **Causes of death selected in Nolte and McKee’s list**[11] |
| --- | --- |
| **Infectious diseases** | Tuberculosis |
|  | Septicaemia |
|  | Pneumonia |
|  | Influenza |
|  | Intestinal infections (other than typhoid, diphtheria) *<14* |
|  | Diphtheria, tetanus, poliomyelitis |
|  | Whooping cough *<14* |
|  | Measles *1–14* |
| **Neoplasms (cancers)** | Colorectal cancer |
|  | Malignant neoplasms of skin |
|  | Breast cancer |
|  | Cervical cancer and uterine cancer *<45* |
|  | Neoplasm of the testis |
|  | Hodgkin’s disease |
|  | Leukaemia *<45* |
| **Endocrine, nutritional and metabolic diseases** | Thyroid disorders |
|  | Diabetes mellitus *<50* |
| **Diseases of the nervous system** | Epilepsy |
| **Diseases of the circulatory system** | Rheumatic heart diseases |
|  | Ischemic heart diseases – 50 % of deaths |
|  | Cerebrovascular diseases |
|  | Hypertensive diseases |
| **Diseases of the genitor-urinary system** | Nephritis and nephrosis |
|  | Benign prostatic hyperplasia |
| **Diseases of the respiratory system** | All respiratory diseases (excl. pneumonia/influenza) *1–14* |
| **Diseases of the digestive system** | Peptic ulcer |
|  | Appendicitis |
|  | Abdominal hernia |
|  | Cholelithiasis and cholecystitis |
| **Perinatal mortality** | Maternal deaths |
|  | Perinatal deaths (excl. stillbirths) |
|  | Congenital cardiovascular anomalies |
| **External causes** | Misadventures to patients during surgical and medical care |

Note: age limit is 75 years except if otherwise mentioned
